# Molecular insights into cell toxicity of a novel familial amyloidogenic variant of β2‐microglobulin

**DOI:** 10.1111/jcmm.12833

**Published:** 2016-03-18

**Authors:** Manuela Leri, Francesco Bemporad, Reinier Oropesa‐Nuñez, Claudio Canale, Martino Calamai, Daniele Nosi, Matteo Ramazzotti, Sofia Giorgetti, Francesco S. Pavone, Vittorio Bellotti, Massimo Stefani, Monica Bucciantini

**Affiliations:** ^1^ Dipartimento di Scienze Biomediche, Sperimentali e Cliniche ‘Mario Serio’Università degli Studi di FirenzeFirenzeItaly; ^2^ Dipartimento di NanofisicaIstituto Italiano di TecnologiaGenovaItaly; ^3^ European Laboratory for Non‐linear Spectroscopy (LENS)Università degli Studi di FirenzeSesto FiorentinoItaly; ^4^ National Institute of OpticsConsiglio Nazionale delle Ricerche (CNR)FirenzeItaly; ^5^ Dipartimento di Medicina Sperimentale e ClinicaUniversità degli Studi di FirenzeFirenzeItaly; ^6^ Dipartimento di Medicina MolecolareIstituto di BiochimicaUniversità degli Studi di PaviaPaviaItaly; ^7^ Wolfson Drug Discovery UnitCentre for Amyloidosis and Acute Phase ProteinsDivision of MedicineRoyal Free Campus University College LondonLondonUK; ^8^ Centro Interuniversitario per lo Studio delle Malattie Neurodegenerative (CIMN)FirenzeItaly

**Keywords:** membrane bilayers, amyloid cytotoxicity, GM1 ganglioside, protein misfolding, systemic amyloidosis

## Abstract

The first genetic variant of β_2_‐microglobulin (b2M) associated with a familial form of systemic amyloidosis has been recently described. The mutated protein, carrying a substitution of Asp at position 76 with an Asn (D76N b2M), exhibits a strongly enhanced amyloidogenic tendency to aggregate with respect to the wild‐type protein. In this study, we characterized the D76N b2M aggregation path and performed an unprecedented analysis of the biochemical mechanisms underlying aggregate cytotoxicity. We showed that, contrarily to what expected from other amyloid studies, early aggregates of the mutant are not the most toxic species, despite their higher surface hydrophobicity. By modulating ganglioside GM1 content in cell membrane or synthetic lipid bilayers, we confirmed the pivotal role of this lipid as aggregate recruiter favouring their cytotoxicity. We finally observed that the aggregates bind to the cell membrane inducing an alteration of its elasticity (with possible functional unbalance and cytotoxicity) in GM1‐enriched domains only, thus establishing a link between aggregate‐membrane contact and cell damage.

## Introduction

β_2_‐microglobulin (b2M) is a 99 residue‐long human protein belonging to the major histocompatibility complex class I (MHC I). The protein acts as a chaperone during the assembly of MHC I quaternary structure, an event that is required for antigen presentation 1. Structurally, b2M is an all‐β protein with seven anti‐parallel β‐strands organized into two β‐sheets and stabilized by a single disulphide bridge 2. In addition to its physiological function, much clinical interest has been drawn to b2M as its increased serum levels and misfolding have been linked to a pathological condition known as dialysis‐related amyloidosis (DRA) 3. Normally, once released from the quaternary structure of MHC I, b2M is cleared in the proximal tubules of the kidneys. However, the serum concentration of b2M in patients undergoing dialysis rises from normal levels (1–2 mg/l) to as much as 50–70 mg/l 3, 4. Enhanced concentration, in conjunction with other factors such as the presence of Cu^2+^ ions 5, 6, collagen 7, glycosaminoglycans 8 and other factors 9, results in b2M misfolding and eventually to the formation of amyloid aggregates that deposit mainly into joints.

Although conversion of wild‐type b2M into amyloid fibrils is difficult to achieve *in vitro* under physiological conditions, many recombinant b2M variants have been investigated, and hypotheses about the mechanisms underlying protein fibrillogenesis have been put forward 3. The proposed aggregation mechanism of b2M involves the formation of an amyloidogenic native‐like conformation, usually referred to as N_T_ state, which, albeit being globally folded, bears the His31‐Pro32 peptide bond in the non‐native *trans* configuration 3, 10. This view has been confirmed by recent data showing that b2M stability, (un)folding and aggregation properties are all influenced by the kinetics and equilibrium of Pro32 *cis*‐*trans* isomerization 11. Until recently, no natural pathological mutations of b2M were recognized. However, a recent report described a French kindred carrying a heterozygous point mutation in the gene encoding b2M 4 which results in the replacement of aspartate 76 with an asparagine (D76N b2M). The pathogenic protein was aggressively fibrillogenic *in vitro* under conditions where the stability of the wild‐type protein is unaffected, prompting a re‐evaluation of previously hypothesized mechanisms of b2M fibrillogenesis. Individuals of this family suffered a complex set of clinical symptoms, including bowel dysfunction with chronic diarrhoea and sicca syndrome, weight loss and postural dizziness 4. ^123^I‐human serum amyloid protein (SAP) scans and postmortem examination of affected people revealed the presence of amyloid deposits in the spleen, liver, heart, peripheral nerves, salivary and adrenal glands. Importantly, the D76N b2M present in amyloid fibrils extracted from patients consisted only of the full‐length variant without any truncated form. Moreover, in contrast to DRA patients, all the members of this family displayed normal circulating concentrations of b2M and normal renal function. Consequently, the pathological condition of these patients was described as a hereditary systemic amyloidosis associated with D76N b2M 4.

The experimental evidence described above points towards a high amyloidogenic potential of D76N b2M. In fact, when incubated *in vitro* at a protein concentrations 20‐fold higher than those found in patients under dialysis, wild‐type b2M remains predominantly monomeric for several months 12. Under these conditions, b2M aggregation can be induced only by the aforementioned cofactors or at low pH and in the presence of co‐solvents such as 2,2,2‐trifluoroethanol 3. By contrast, D76N b2M rapidly aggregates when incubated at 37°C at physiological pH and ionic strength; under these conditions, the aggregation can be accelerated by agitation that enhances the air–water interface. Atomic force microscopy images showed that amyloid fibrils grown from D76N b2M are already present after 8 hrs since the beginning of the aggregation process 10. The crystal structure of the D76N variant at 1.40 Å resolution provided clues to explain its reduced stability and its increased fibrillogenic potential. The amide group of Asn76 establishes a new hydrogen bond with Tyr78, which moves closer to residue 76 providing a hydrogen bond to the amide nitrogen of Thr73. In addition, the theoretical isoelectric point of the mutant shifts from 6.05 to 6.40 due to the loss of the negative charge of Asp76 4. In an attempt to get mechanistic insight into the aggregation properties of D76N b2M, it was found that although the solution structure of the mutant does not differ significantly from that of the wild‐type protein, such variant is strongly destabilized when compared with the latter, with denaturation free energy values of 5.7 ± 0.4 and 3.00 ± 0.15 kcal/mol for wild‐type and D76N b2M, respectively 10. Such destabilization arises from accelerated unfolding and slower refolding. NMR experiments were consistent with such measurements, revealing that D76N b2M possesses enhanced molecular dynamics and loss of rigidity. Intriguingly, a set of experiments on folding/unfolding kinetics showed that the amyloidogenic N_T_ state is significantly more populated in the variant. Indeed, the *in vitro* equilibrium concentration of such conformation, measured at physiological pH and in the absence of denaturants, appears increased in the case of the variant, with values of 4.8 ± 3.0% and 25 ± 9.0% for wild‐type and D76N b2M, respectively 10.

Although the pieces of evidence described above provide valuable information concerning the *in vitro* stability and aggregation propensity of D76N b2M, additional information is still required to link these data to amyloid‐related toxicity of this variant. In this article, we exploited a battery of complementary biophysical tools to characterize the species transiently populated during D76N aggregation. The ability of such aggregates to interact with model membranes bearing different lipid composition was also evaluated. The outcome of these experiments was compared with cell biology studies aimed at investigating the mechanisms of cytotoxicity of the different aggregation states of D76N b2M. These results provide an unprecedented characterization of the pathway spanning from protein misfolding to the cell dysfunctions underlying b2M hereditary systemic amyloidosis.

## Materials and methods

### Preparation of D76N aggregates

D76N b2M was expressed and purified as previously reported 4, 10. The aggregation reaction was initiated starting from the freeze‐dried protein. Briefly, the protein was dissolved in PBS (Sigma‐Aldrich, St. Louis, MO, USA). At time 0 of the aggregation reaction, the protein solution was centrifuged for 5 min. at 13,200 × g and filtrated through 0.02 μm nanofilters (Whatman Inc., Amersharm, UK) to eliminate possible aggregation seeds. The resulting supernatant was diluted to a protein concentration of 20 μM. The latter was determined by measuring the absorption at 280 nm, using a molar extinction coefficient of 19,940 per M/cm, a 1.0‐mm path‐length cell and a Jasco V‐630 UV‐visible spectrophotometer (Tokyo, Japan). A 300 μl of the obtained solution was incubated in a 1.5‐ml eppendorf tube lying in horizontal position, under vigorous shaking at 37°C.

### Far‐UV circular dichroism spectroscopy

A far‐UV circular dichroism (CD) spectrum of 0.2 mg/ml D76N b2M was acquired at 25**°**C in PBS. The spectrum was collected over the 190–250 nm wavelength range using a Jasco‐810 Spectropolarimeter equipped with a thermostated cell holder attached to a Thermo Haake C25P water bath (Karlsruhe, Germany). A 1.0‐mm path‐length cell was used. The spectrum was blank subtracted and converted to mean residue ellipticity per residue (θ).

### Dinamic light scattering

Size distribution analysis was carried out at 25°C using a Malvern Zetasizer Nano S dynamic light scattering (DLS) device (Malvern, Worcestershire, UK) on 20 μM D76N b2M samples. Each sample was analysed considering the refraction index and viscosity of its dispersant. A 10‐mm reduced volume plastic cell was used. The acquired data were compared with the theoretical hydrodynamic radius expected for folded polypeptide chains, calculated as *R*_*h*_^folded^ = 4.92*N* ^0.285^, where *N* is the number of residues and *R*_*h*_^folded^ is the hydrodynamic radius expressed in Å 13.

### Congo Red absorbance

Congo Red (CR; Sigma‐Aldrich) spectra were recorded at 25°C in the presence of D76N b2M. For any time‐point, three spectra were acquired containing (*i*) 60 μl of the aggregating protein solution and 440 μl of a solution containing 20 μM CR (spectrum 1), (*ii*) 60 μl of PBS devoid of protein and 440 μl of buffer containing 20 μM CR (spectrum 2) and (*iii*) 60 μl of protein and 440 μl of PBS in the absence of CR (spectrum 3). The spectra were recorded from 400 to 700 nm using a Jasco V‐630 spectrophotometer and a 5.0‐mm reduced volume quartz cell. The difference spectra were obtained by subtracting spectrum 2 and spectrum 3 from spectrum 1.

### Thioflavin T fluorescence

Aliquot of 62 μl of the aggregation solution was mixed with 438 μl of a solution containing 25 μM thioflavin T ThT dissolved in a 25 mM phosphate buffer, pH 6.0. Fluorescence spectra from 450 to 600 nm were acquired at 25°C in a 2 × 10 mm quartz cuvette using a Perkin‐Elmer LS 55 spectrofluorimeter (Waltham, MA, USA) equipped with a thermostated cell holder attached to a Haake F8 water bath. Excitation wavelength was 440 nm. The ThT fluorescence spectrum obtained under the same conditions without D76N b2M was subtracted from that acquired in the presence of the dye. The data were fitted to an empirical sigmoid function 14:

(1) $$F=F_{0}+A/\left\{1+\text{exp}[k_{\text{agg}}\left(t_{1/2}-t\right)]\right\}$$

where *F* is the measured fluorescence, *F*_0_ is the fluorescence at the beginning at the reaction, *t*_1/2_ is the midpoint of the reaction, *k*_agg_ is the apparent aggregation rate and *A* is the jump in fluorescence upon aggregation. The length of the lag‐phase *t*_lag_ can be calculated as *t*_lag_ = *t*_1/2_ − 2/(*k*_agg_) 14.

### ANS fluorescence

Samples containing aggregating D76N b2M at 20 μM were investigated for their ability to bind 8‐anilinonaphthalene‐1‐sulfonic acid (ANS; Sigma‐Aldrich). Briefly, 100 μl of the aggregation solution were mixed with 500 μl of a solution containing 120 μM ANS in PBS. Then, the fluorescence spectra were recorded in the 400–600 nm range using the same equipment and cuvette described above for the ThT fluorescence experiments. Excitation wavelength was 370 nm. The data were analysed by subtracting blank spectra consisting of the dye in PBS without protein.

### Transmission electron microscopy

Transmission electron microscope (TEM) imaging was carried out as follows. Briefly, 5.0‐μl aliquots of D76N b2M were withdrawn at different aggregation times, loaded onto a formvar/carbon‐coated 400 mesh nickel grids (Agar Scientific, Stansted, UK) and negatively stained with 2.0% (w/v) uranyl acetate (Sigma‐Aldrich). The grid was air‐dried and examined using a JEM 1010 TEM at 80‐kV excitation voltage.

### Cell culture and cell viability assay

The SH‐SY5Y cells were cultured at 37°C in complete medium [50% HAM, 50% DMEM, 10% foetal bovine serum (FBS), 3.0 mM glutamine, 100 units/ml penicillin and 100 μg/ml streptomycin], in a humidified, 5.0% CO_2_ incubator. All the materials used for cell culture were from Sigma. HL‐1 mouse atrial myocytes were obtained from Dr W. C. Claycomb (Louisiana State University Health Science Center, New Orleans, LA, USA) and grown in T25, gelatin/fibronectin‐coated flasks, as previously described 15. The cells were maintained in Claycomb Medium (SAFC, Saint Louis, MO, USA) supplemented with 10% FBS containing 2.0 mM L‐glutamine, 0.1 mM noradrenaline and 100 U/ml penicillin‐streptomycin (Sigma‐Aldrich). Every 3 days, the cells (70–90% confluent) were detached and re‐plated at a 1:3 dilution in a new T25 flask or in 96‐well plates and used for measurements. Samples containing aggregating D76N b2M were administered to cells at a final concentration of 5.0 μM. The toxicity of the different forms of D76N b2M aggregates was assessed by the 3‐(4,5‐dimethylthiazol‐2‐yl)‐2,5‐diphenyltetrazolium bromide (MTT) (Sigma‐Aldrich) reduction inhibition assay based on the protocol described for the first time by Mosmann 16. In all MTT experiments, the cells were plated at a density of 10,000 cells per well on 96‐well plates in 100‐μl culture medium. After 48 hrs, the cells were treated with the aggregates for 24 hrs and then incubated for a further 2.0 hrs with 100 μl of DMEM without phenol red, containing 0.5 mg/ml MTT. After 2.0 hrs, 100 μl of cell lysis buffer (20% SDS, 50% *N*,*N*‐dimethylformamide, pH 4.7) was added to each well and the samples were incubated at 37°C to allow complete lysis. The absorbance values of blue formazan were determined at 595 nm with an automatic plate reader (Bio‐Rad, Milan, Italy). The final absorption values were calculated by averaging three independent measurements of each sample and subtracting from this the average of the blank solution, consisting of 100 μl of MTT solution and 100 μl of lysis buffer (20% SDS, 50% *N*,*N*‐dimethylformamide). All data were expressed as mean ± S.D.

### Reactive oxygen species measurement

The intracellular levels of reactive oxygen species (ROS) were determined using the fluorescent probe 2′,7′–dichlorofluorescin diacetate, acetyl ester (CM‐H_2_ DCFDA; Sigma‐Aldrich). CM‐H_2_ DCFDA is a cell‐permeant indicator for ROS that is not fluorescent until removal of the acetate groups by intracellular esterases and subsequent oxidation. The latter can be detected by monitoring the increase in fluorescence at 538 nm. The SH‐SY5Y and HL‐1 cells were plated at a density of 10,000 cells per well on 96‐well plates. After 24 hrs of cell exposure to the aggregates, 10 μM DCFDA in DMEM without phenol red was added. After 30 min, the fluorescence values at 538 nm were detected by Fluoroscan Ascent FL (Thermo‐Fisher, Illkinch, France).

### Cytosolic calcium levels

The cytosolic levels of free Ca^2+^ were measured using the fluorescent probe Fluo‐3 acetoxymethyl ester (Fluo‐3 AM; Invitrogen, Monza, Italy). Subconfluent SH‐SY5Y cells cultured on glass coverslips were incubated at 37°C for 5.0 min with 5.0 μM Fluo‐3 AM prior to exposure to D76N b2M aggregates for 1.0 hr. At the end of the incubation, the cells were fixed in 2.0% buffered paraformaldehyde for 10 min. Cell fluorescence was visualized using a confocal Leica TCS SP5 scanning microscope (Leica, Mannheim, Germany) equipped with a HeNe/Ar laser source for fluorescence measurements. The observations were performed using a Leica Plan 7 Apo X63 oil immersion objective, suited with optics for DIC acquisition. Cells from five independent experiments and three areas (about 20 cells/area) per experiment were analysed. The fluorescence intensity of Fluo3‐AM was analysed by using ImageJ software (National Institutes of Health, Bethesda, MD, USA) and expressed as arbitrary units.

### Cell death detection

Cell apoptosis was detected by the Annexin V‐fluorescein isothiocyanate (FITC) Apoptosis detection kit (Sigma‐Aldrich). A propidium iodide (PI) solution containing Annexin V‐FITC was used to discriminate viable from apoptotic and secondary necrotic cells. Briefly, after a 24‐hr treatment with the most toxic D76N b2M aggregates, the cells were incubated with Annexim V‐FITC and PI for 10 min, at room temperature. Then, the cells were analysed by flow cytometry. Annexin V‐FITC was detected as a green fluorescence at 514 nm and PI was detected as a red fluorescence at 617 nm.

### Confocal immunofluorescence

Subconfluent SH‐SY5Y and HL‐1 cells grown on glass coverslips were treated for 24 hrs with the different D76N b2M aggregates grown for various time lengths, at 5.0 μM final concentration. After incubation, the cells were washed with PBS; then, GM1 labelling at the cell surface was performed by incubating the cells with 10 ng/ml CTX‐B Alexa488 in complete medium for 10 min at room temperature. Finally, the cells were fixed in 2.0% buffered paraformaldehyde for 10 min., permeabilized by treatment with a solution containing 50% acetone and 50% ethanol for 4.0 min. at room temperature, washed with PBS and blocked with PBS containing 0.5% BSA and 0.2% gelatine. After blocking, the cells were incubated for 1.0 hr at room temperature with a rabbit polyclonal antibody raised against b2M (Abcam, Cambridge, UK) diluted 1:600 in the blocking solution and washed with PBS for 30 min. under stirring. The immunoreaction was revealed with Alexa 568‐conjugated anti‐rabbit secondary antibody (Invitrogen) diluted 1:100 in PBS. Finally, the cells were washed twice in PBS and once in water to remove non‐specifically bound antibodies. Cell fluorescence was visualized using a confocal Leica TCS SP5 scanning microscope (Leica) equipped with a HeNe/Ar laser source for fluorescence measurements. The observations were performed using a Leica Plan 7 Apo X63 oil immersion objective, suited with optics for DIC acquisition.

### GM1 depletion

To reduce cell membrane GM1, the glucosylceramide synthase of the plated cells was inhibited by supplementing the cell culture medium with 25 μM D‐threo‐1‐phenyl‐2‐decanoylamino‐3‐morpholino‐1‐propanol (PDMP; Matreya, LLC, Bellefonte, PA, USA) for 72 hrs at 37°C in complete medium as previously described 17. The same pre‐treatment was used for cells subjected both to MTT assay and to immunofluorescence imaging. Cells from five independent experiments and three areas (about 20 cells/area) per experiment were analysed. The fluorescence intensity of CTX‐B Alexa488 was analysed by using ImageJ software (National Institutes of Health) and expressed as arbitrary units.

### Single particle imaging and tracking

Quantum dots (QDs) labelling and live imaging have been extensively described 18. Briefly, living cells previously exposed to D76N b2M aggregates were incubated at 37°C in phenol red‐free Leibovitz's L‐15 medium 10% FBS with anti‐b2M antibodies (1:1000) for 20 min., then for 5.0 min. with antimouse Alexa 488 (1:500) and 10 μg/ml biotinylated CTX‐B and finally with streptavidin QDs (Invitrogen) in QD binding buffer for 1.0 min. QDs emitting at 655 nm were used at a 1:10,000 dilution. The cells were monitored with a custom‐made wide‐field epifluorescence microscope equipped with an oil‐immersion objective (Nikon Plan Apo TIRF 60×/1.45), a Reliant 150 Select argon ion laser (excitation line 488 nm) and a heating chamber. A FF499‐Di01‐25 dichroic and FF01‐655/15‐25 (for QDs) and FF01‐530/43‐25 (for Alexa 488) emission filters (Semrock) were used; 250 or 100 consecutive frames were acquired with an integration time of 10 msec., with an Electron Multiplying Charge‐Coupled iXon Ultra camera (Andor, Belfast, UK). Recording sessions did not last more than 30 min. Tracking of single QDs, identified according to their fluorescence intermittence, was performed with MATLAB (MathWorks, Natick, MA, USA) using a macro that accounts for blinking in the fluorescence signal 18, 19, 20. In brief, the method consisted of two main steps, applied consecutively to each frame of the sequence. First, the fluorescent spots were detected by cross‐correlating the image with a Gaussian model of the Point Spread Function. A least‐squares Gaussian fit was applied (around the local maximum above a threshold) to determine the centre of each spot with a spatial accuracy of 10–20 nm (depending on the signal‐to‐noise ratio). Second, QD trajectories were assembled automatically by linking, frame by frame, the centres of the fluorescent spots likely coming from the same QD. The association criterion was based on the assumption of free Brownian diffusion and took into account short blinking events. After processing, a manual association step was performed, in which QD trajectories of maximal length were assembled from smaller fragments separated by longer blinking events that were not taken into account by the automatic linking procedure. A high concentration of pentavalent B subunit of cholera toxin can in principle induce cross‐linking of GM1. For SPT experiments, however, we incubated the cells with CTX‐B for times shorter than for standard immunolabelling experiments, thus obtaining a lower level of labelling. The concentration of strep‐QDs was largely in excess with respect of biotin‐CTX‐B. Most of, if not all, the CTX‐B molecules bound to the plasma membrane are therefore expected to be labelled.

### Quantitative analysis of diffusion coefficient

The mean square displacement (MSD) analysis allows to calculate the initial diffusion coefficient (D) of each particle 18, 19, 20, 21. Briefly, physical parameters can be extracted from each trajectory (*x*(*t*), *y*(*t*)) by computing the MSD 22, determined from the following formula:

$$\text{MSD}\left(ndt\right)=\frac{1}{N-n}\sum_{i=1}^{N-n}\left[{\left(X_{\left(i+n\right)}-X_{i}\right)}^{2}+{\left(Y_{\left(i+n\right)}-Y_{i}\right)}^{2}\right]$$

where *x*_*i*_ and *y*_*i*_ are the coordinates of a particle on frame *i*,* dt* is the time between two successive frames, *N* is the total number of frames of the trajectory and *ndt* is the time interval over which the displacement is averaged. This function enables the analysis of the lateral dynamics on short‐ (initial diffusion coefficient) and long‐ (types of motion) time scales. Different types of motion can be distinguished from the time dependence of the MSD 21. The initial diffusion coefficient (D) is determined by fitting the initial 2–5 points of the MSD against time plot with MSD(*t*) = 4D_2‐5_ *t* + b. The cumulative probability C(*d*) of D defines the probability that D is less than *d*. We compared cumulative probability distributions and median instead of mean values because D values were spread over four orders of magnitude. The Images were thresholded with ImageJ software, creating binary masks corresponding to amyloid aggregates.

### Synthetic lipid vesicle preparation

1,2‐dioleoyl‐sn‐glycero‐3‐phosphocholine (DOPC,18:1), sphingomyelin (SM) (brain, porcine) and ganglioside GM1 (brain, ovine‐ sodium salt) were purchased from Avanti Polar Lipids (Alabaster, Alabama, USA); cholesterol (Chol), chloroform and methanol were purchased from Sigma‐Aldrich. Two different mixtures were prepared, containing DOPC:SM at 2:1 molar ratio, a fixed, 1.0%, molar percentage of cholesterol and a variable percentage of 5.0% GM1, when present. To prepare the liposomes in solution, we dissolved the phospholipid powder in chloroform/methanol (2:1) according the desired composition and gently evaporated the mixture to dryness under a nitrogen flux. Aliquots were stored overnight under vacuum and resuspended in Milli‐Q water to form multilamellar vesicles. Multilamellar vesicles suspensions were pre‐sonicated at 60°C for 1.0 hr and then extruded 11 times through polycarbonate membrane with 100 nm pores using a commercial extruder (Avanti Polar Lipids) at 60°C. The suspensions were left to cool at room temperature and the large unilamellar vesicles suspensions so obtained were diluted 10‐fold in Milli‐Q water; then 40 μl of each suspension and 10 μl of 10 mM CaCl_2_ solution were deposited on a 1.0 cm × 1.0 cm freshly cleaved mica substrate. In order to get uniform bilayer coverage, the samples were incubated for 15 min. at 60°C after 10 min. storage at room temperature; then the samples were kept at room temperature in a close chamber with 100% relative humidity. Before atomic force microscopy (AFM) measurements, SLBs were gently rinsed three times with Milli‐Q water to remove excess vesicles from the liquid subphase.

### AFM imaging and elasticity measurements

AFM images and indentation measurements were performed by using a Nanowizard III (JPK Instruments, Berlin, Germany) mounted on an Axio Observer D1 (Carl Zeiss, Wetzlar, Germany). inverted optical microscope. V‐shaped DNP silicon nitride cantilevers (Bruker, Billerica, MA, USA), with a nominal spring constant 0.06 N/m, resonance frequency in air in the 40–70 kHz range and tip typical curvature radius of 20–60 nm were used. The actual spring constant of each cantilever was determined *in situ*, using the thermal noise method. D76N b2M aggregates at different times of aggregation (1, 24, 72 and 144 hrs) were administered to the sample under the AFM head at a 5.0‐μM final concentration and let incubating for 20 min. before further measurements. Before injection, the AFM tip was lifted up 60 μm from the sample. Quantitative Imaging mode (QI–JPK Instruments) was used to determine sample topography and elasticity (Young's Modulus) before and after the administrations of the protein aggregates. QI is based on the acquisition of a large set of force–distance curves and on the reconstruction of sample topography from the tip position at the specific force load. Local mechanical properties of the sample can be extracted from the analysis of the acquired curves. QI images deriving from 128 × 128 force–distance (FD) curves were acquired on different samples before and after protein administration, with maximum force load of 1.0 nN. For each curve, the tip speed was 25 μm/sec. and the curve length was 100 nm. For absolute value determination of the Young's Modulus, each single FD curve was fitted with the Hertz model, the tip was approximated as spherical and with a radius of 30 nm. Comparisons between the different cumulative distributions were performed by the Kolmogorov–Smirnov test. A *P*‐value <0.05 was considered statistically significant. SPT data were collected from 10 cells from three independent experiments.

## Results

### D76N b2M aggregation and biophysical characterization of the aggregates

**Figure 1:**
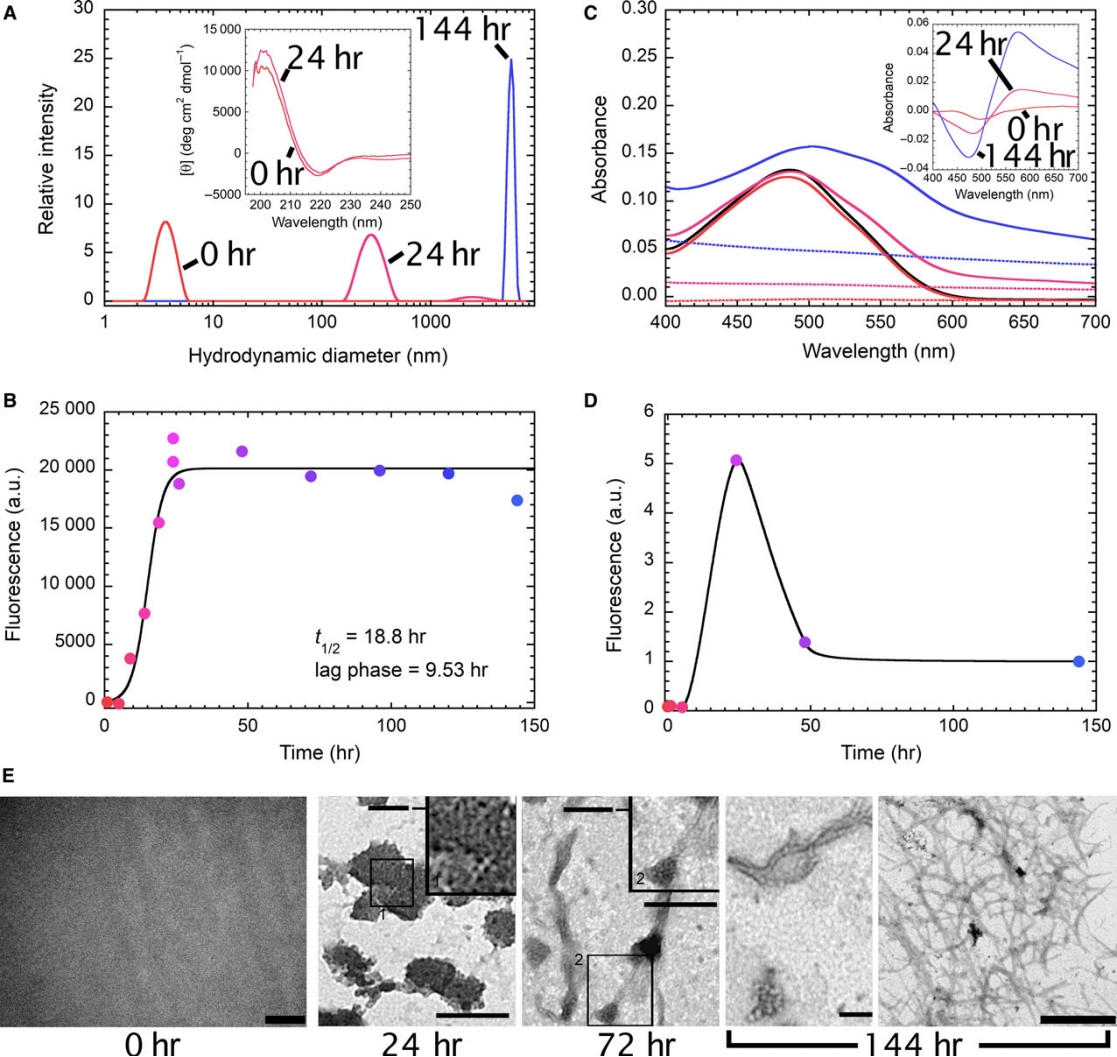
Biophysical characterization of *in vitro* D76N b2M aggregation. In all panels, the colour ranges from red (0 hr) to blue (144 hrs) as time proceeds since the beginning of the aggregation reaction. (**A**) Size distributions of D76N b2M at three different time intervals: 0 hr (red), 24 hrs (magenta) and 144 hrs (blue). The inset shows the far‐UV CD spectrum recorded for the sample at 0 hr and 24 hrs. (**B**) Aggregation of D76N b2M followed by means of ThT fluorescence. The continuous line represents the best fit of experimental data to Equation (1). (**C**) CR absorption spectra in the presence of aggregating D76N b2M after 0 hr (red), 24 hrs (magenta) and 144 hrs (blue) since the beginning of the aggregation reaction. In each case, the continuous line represents CR absorption in the presence of the protein, whereas the dashed line represents protein scattering in the absence of CR. The black continuous line represents CR absorption in the absence of the protein. The inset shows the difference spectra. (**D**) ANS fluorescence during aggregation of the protein. (**E**) TEM images of samples containing D76N b2M after 24, 72 and 144 hrs since the beginning of the aggregation. Bar 0 hr: 0.5 μm; bars 24 and 72 hrs: 200 nm; inset 1 = 50 nm; inset 2 = 100 nm; bars 144 hrs: (a) 200 nm, (b) 1 μm.

Given the high amyloidogenic propensity of D76N b2M, *in vitro* aggregation of the protein was initiated at 37°C in PBS at a concentration of 20 μM under vigorous shaking. Aliquots of the mixture were withdrawn at different time intervals since the beginning of the aggregation reaction, and a number of biophysical properties of the aggregates found in the mixture were investigated (Fig. 1). At the beginning of the aggregation reaction, D76N b2M exhibited a hydrodynamic diameter of 3.7 ± 0.5 nm, as assessed by DLS measurements (Fig. 1A). This value is in agreement with that (3.64 nm) expected for a compactly folded monomeric protein of 99 residues 13. The CD spectrum of the sample showed a single negative peak at 218 nm, confirming the presence of a prevalently native‐like β‐fold, which was retained during the first 24 hrs since the beginning of aggregation (Fig. 1A, inset). Furthermore, at the beginning of the reaction, the protein was unable to bind amyloid reporting dyes, such as Thioflavin‐T (ThT, Fig. 1B) and Congo Red (CR, Fig. 1C) as well as ANS (Fig. 1D). Transmission electron microscopy images confirmed the absence of any aggregated material at the beginning of the reaction (Fig. 1E). However, ThT fluorescence measurements showed that, after a lag‐phase of ca. 9.5 hrs, the emission of the dye in the presence of the aggregating D76N b2M increased, reaching a plateau after around 36 hrs (Fig. 1B). A 18.8‐hr half‐time, as estimated from best fits of experimental data to Equation (1), was calculated for the process. The spectroscopic and microscopic investigation and the DLS measurements of the species populated at 24 hrs revealed a heterogeneous population of multimers with hydrodynamic radii in the 100–300 nm range (Fig. 1A) in the absence of any fibrillar component (Fig. 1E); these assemblies were able to bind ThT (Fig. 1B), ANS (Fig. 1D) and, in part, CR as confirmed by the presence of the peak at 570 nm (Fig. 1C). Fibrils started to appear after 72 hrs of incubation under our aggregation conditions (Fig. 1E) and the species populated at 144 hrs reached the highest values of hydrodynamic diameter (Fig. 1A). These fibrils were still able to bind ThT (Fig. 1B) and exhibited a marked increase in CR binding (Fig. 1C) and a decrease in ANS binding (Fig. 1D). At these time points, the TEM images revealed the presence of bundles of unbranched fibrils with a diameter of about 20 nm (Fig. 1E a, b). Fibril formation after 72 hrs made it impossible to record CD spectra for such species, because light scattering by protein aggregates made the spectra too noisy (data not shown). Taken together, these data show lead to conclude that, under our conditions, D76N b2M converts from an initial fully soluble, folded and monomeric state into insoluble fibrillar assemblies exhibiting tinctorial properties typical of amyloid aggregates. The process occurs *via* the transient formation of self‐assemblies that bind ThT and ANS and display solvent‐exposed hydrophobic patches responsible for the formation of large clusters (Fig. 1E).

### D76N aggregates induce alteration of Ca^2+^ and ROS levels leading to necrotic death

**Figure 2:**
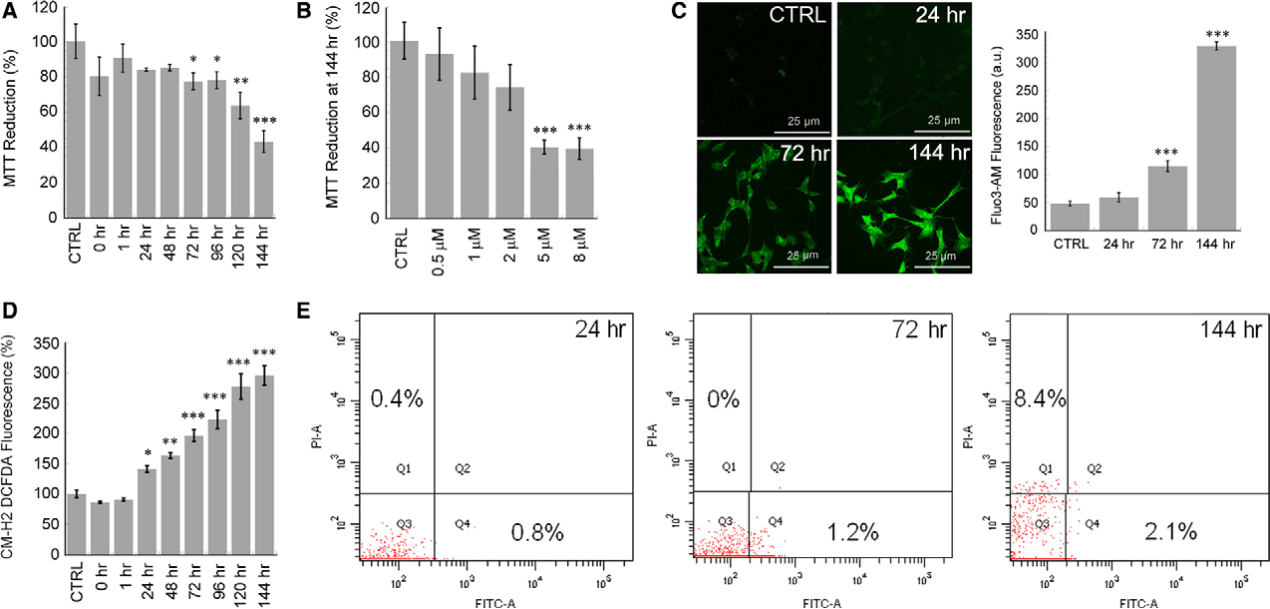
Cytotoxicity of D76N b2 m aggregates. (**A**) MTT assay of SH‐SY5Y cells exposed for 24 hrs to 5.0 μM D76N b2M samples aggregated for different times. (**B**) Dose‐dependent effect of 144‐hr‐aged D76N b2M on SH‐SY5Y viability. (**C**) Confocal microscopy imaging of intracellular free Ca^2+^ levels in SH‐SY5Y cells exposed for 1.0 hr to 5.0 μM D76N samples aggregated for 24, 72 or 144 hrs. Right: quantification of Ca^2+^ levels with respect to untreated cells. (**D**) ROS production in SH‐SY5Y cells exposed for 24 hrs to D76N samples (5.0 μM) aggregated for varying lengths of time. Error bars in all bar plots indicate the standard deviation of three independent experiments carried out in triplicate. *T*‐test analysis: *P < 0.005; **P < 0.001;***P < 0.0001 *versus* untreated cells. (**E**) FACS analysis of cells treated with D76N b2M aggregates aged 24, 72 or 144 hrs. The plots show the scatter dot plots of Annexin V FITC‐A *versus* propidium iodide. The percentages of gated cells in the two populations are also reported.

We also investigated whether the species transiently populated along the aggregation path of D76N b2M were toxic and, if so, whether toxicity was associated to membrane binding and/or aggregate internalization. The cytotoxicity in our cell model was studied using human SH‐SY5Y neuroblastoma cells previously shown to be sensitive to the toxicity of 20 μM b2M aggregates 23. To this aim, human SH‐SY5Y cells were exposed for 24 hrs to 5.0 μM D76N b2M aggregates (monomer protein concentration) grown for different times under our aggregation conditions; then, aggregate cytotoxicity was evaluated by the MTT assay. When compared to the exposure of cells to monomeric D76N b2M, the cytotoxicity of early aggregates was very low; significant toxicity values started to appear in cells exposed to aggregates aged 72 hrs and reached the highest values in cells exposed to samples aged 120 or 144 hrs (Fig. 2A). In addition, the significant cytotoxic effect of 144‐hr sample on SH‐SY5Y cells resulted at the concentration of 5.0 μM and 8.0 μM (Fig. 2B). These data match the presence of fibrillar material, suggesting that cytotoxicity is associated with fibrils. Our data agree with previous studies showing a dose dependence of the cytotoxic effect of the wild‐type b2M in the 1‐ to 20‐μM concentration range and an IC50 value around 4.6 μM 23. In addition, comparable levels of cytotoxicity were observed for early aggregates of D76N b2M and wild‐type b2M oligomers 23. Similar levels of cytotoxicity were observed on the mouse cardiac cell line HL‐1 treated for 24 hrs with 5.0 μM D76N b2M aggregates (Fig. S2C) reinforcing the role of this b2M variant in the onset of a systemic amyloidosis 4. Next, we sought to provide information on the mechanisms of cell damage following exposure to toxic D76N b2M aggregates by measuring intracellular free Ca^2+^ and ROS levels using the fluorescent probes Fluo‐3‐acetoxymethyl ester (Fluo‐3AM) and CM‐H_2_DCFDA, respectively. Figure 2C shows a twofold and a sevenfold increase of the intracellular Ca^2+^ concentration in cells treated for 1.0 hr with 5.0 μM D76N b2M aggregates aged 72 or 144 hrs, respectively, whereas no significant changes were observed when the cells were treated for 1.0 hr with vehicle (PBS) or with protein samples obtained at early aggregation times (up to 24 hrs), in agreement with the MTT data. Since derangement of intracellular free Ca^2+^ levels and ROS production are biochemically related parameters, we also measured the amount of ROS levels in SH‐SY5Y cells treated with D76N b2M samples aggregated for different time periods. Figure 2D shows that ROS levels increased in cells exposed to increasingly aged D76N b2M. The highest ROS increase (3.5‐fold with respect to untreated cells) was observed after cell treatment with the most toxic, 144‐hr‐aged aggregates, in agreement with the free Ca^2+^ and MTT results. The same results were obtained with HL‐1 cells (Fig. S2D) confirming that the 144‐hr‐aged aggregates were the most aggressive form.

Finally, we investigated whether the aggregate‐exposed cells underwent death and whether the latter was of necrotic or apoptotic type, considering that apoptosis and necrosis can both be triggered by amyloid aggregates 24. SH‐SY5Y cells were treated for 24 hrs with the most toxic, 144‐hr‐aged, D76N b2M aggregates and then labelled with FITC‐conjugated Annexin V, a specific dye that detects phosphatidylserine externalization (as a marker of apoptosis), and PI that labels the cellular DNA in necrotic cells. Such a double labelling allowed us to differentiate early apoptotic cells (Annexin V‐positive, PI‐negative), necrotic cells (Annexin V‐negative, PI‐positive) and fully viable cells (Annexin V‐negative, PI‐negative). Figure 2E shows that 8.4% of the cells treated with the aggregates were Annexin V‐negative but IP‐positive, confirming the occurrence of necrotic death rather than the induction of the apoptotic pathway.

### Interaction with the cell membrane is required for aggregate cytotoxicity

**Figure 3:**
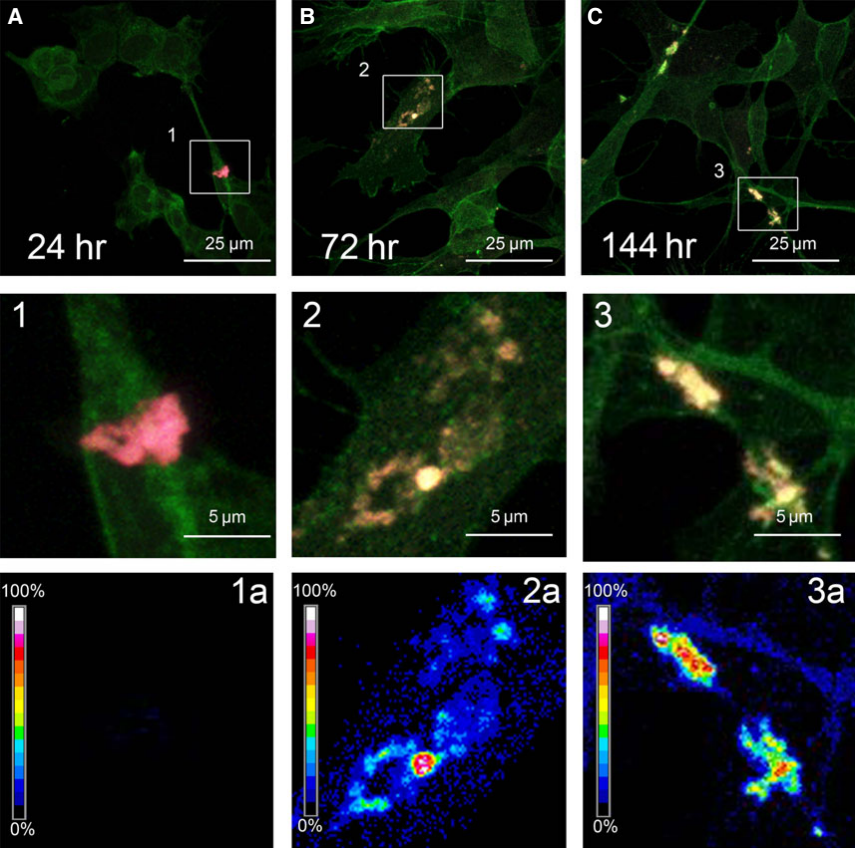
Immunolocalization of D76N b2M aggregates onto the plasma membrane. SH‐SY5Y cells exposed for 24 hrs to 5.0 μM D76N b2M aggregated for 24 hrs (**A**), 72 hrs (**B**) or 144 hrs (**C**). The cells were stained with Alexa 488‐conjugated CTX‐B (green fluorescence); protein aggregates were stained with anti‐b2M antibodies followed by treatment with Alexa 568‐conjugated anti‐rabbit secondary antibodies (red fluorescence). FRET efficiency is shown in **1a**,** 2a**,** 3a** for aggregates aged 24, 72 or 144 hrs, respectively.

It is widely reported that a main mechanism of amyloid aggregate cytotoxicity requires the primary interaction between the aggregates and the cell membrane, resulting in functional and/or structural perturbation of the latter. To investigate the ability of variously aged D76N b2M aggregates to interact with the cell membrane of the exposed cells, we performed confocal microscopy experiments using both a polyclonal antibody raised against recombinant b2M and Alexa 488‐conjugated CTX‐B, a probe specific for the monosialotetrahexosylganglioside 1 (GM1), a common lipid raft marker widely reported as a key interaction site for amyloids 25, 26, 27, 28. We imaged the presence of clusters of all aggregates onto the plasma membrane of SH‐SY5Y cells (Fig. 3A–C), suggesting co‐localization with GM1. A FRET analysis was performed to confirm the interaction between GM1 (green) and aggregates (red). However, we observed a high FRET efficiency, particularly for the 144‐hr‐aged aggregates (Fig. 3, 3a), whereas the FRET was completely absent in samples aggregates for 24 hrs (Fig. 3, 1a), suggesting a different way of interaction with the cell membrane of differently aged aggregates. It was not evident any internalization of 144‐hr‐aged aggregates in cells exposed for 24–48 hrs (data not shown), indicating that the toxic effect of these aggregates occurs without internalization. The GM1‐D76N b2M interaction was confirmed also with HL‐1 cells (Fig. S2A and B) treated with 24‐ or 144‐hr‐aged samples. We observed a high efficiency of FRET also for sample aged 24 hrs, confirming the multisystem onset of the disease *in vivo* 4. This is in agreement with the different cell susceptibility to toxic protein aggregates based on the efficiency of different cell types to accumulate amyloid precursors at their plasma membrane 29.

### GM1 depletion decreases the toxicity of D76N b2M aggregates

**Figure 4:**
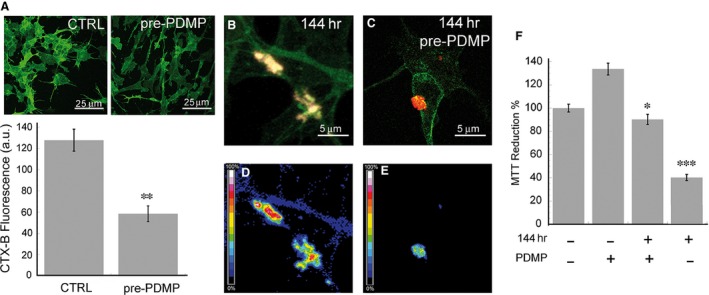
GM1 levels are associated with cell susceptibility to D76N b2M aggregate toxicity. (**A**) Confocal imaging of SH‐SY5Y cells untreated or treated with PDMP and stained with CTX‐B/Alexa 488 (top) and quantification of the corresponding fluorescence signal (bottom) (**B**–**E**) Immunolocalization (**B** and **C**) efficiency of FRET between CTX‐B/Alexa 488 (GM1) and anti‐b2M/Alexa 568 (**D** and **E**) in cells pre‐treated for 72 hrs with 25 μM PDMP (**C** and **E**) and then exposed for 24 hrs to D76N aggregates aged 144 hrs. (**F**) MTT assay carried out with SH‐SY5Y cells pre‐treated for 72 hrs with PDMP and then exposed for 24 hrs to 5.0 μM aggregates aged 144 hrs. Error bars indicate the standard deviation of three (**F**) or five (**A**) independent experiments. *T*‐student analysis: *P < 0.005; **P < 0.001; ***P < 0.0001 *versus* pre‐treated with PDMP (**F**) or *versus* untreated (**A**) cells.

In another set of experiments, we sought to confirm that aggregate toxicity resulted from their ability to interact with the cell membrane at GM1‐enriched sites and hence was dependent on the presence of GM1 in the membrane itself. To this purpose, we modified the ganglioside content in the membrane by pre‐treating the cells with 25 μM PDMP, a glucosylceramide synthase inhibitor that blocks the natural synthesis of GM1 17. The resulting reduction by about twofold of the GM1 content in the plasma membrane (Fig. 4A) left cell viability unaffected (Fig. 4F). As expected, aggregate binding to GM1‐depleted cells exposed for 24‐ to 144‐hr‐aged D76N b2M aggregates was significantly reduced (Fig. 4B and C), as confirmed by the reduced FRET efficiency (Fig. 4D and E); moreover, cell vulnerability to the aggregates was also suppressed (Fig. 4F). These data lend further support to the importance of GM1 as a major membrane binding site for amyloid fibrils 25.

### D76N b2M amyloid aggregates alter GM1 lateral diffusion

**Figure 5:**
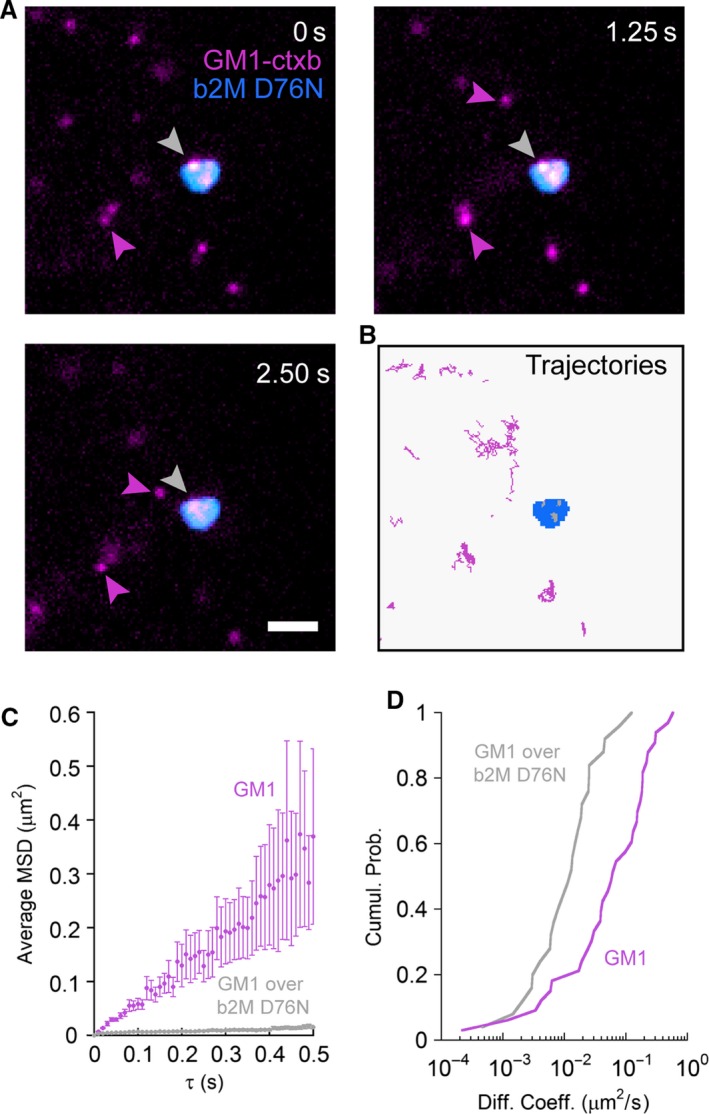
D76N aggregates affect GM1 mobility in living SH‐SY5Y neuroblastoma cells. (**A**) Imaging of single GM1 molecules labelled with biotin‐CTX‐B coupled to streptavidin‐QD 655 (magenta) and 144‐hr‐aged D76N b2M aggregates labelled with anti‐b2M and secondary Alexa 488‐conjugated antibodies (cyan). The scale bar corresponds to 2.0 μm. (**B**) Trajectories of the GM1 molecules in the proximity of (magenta), or overlapping (grey) the D76N b2M aggregates (cyan). (**C** and **D**) Average mean square displacement and cumulative probability distributions of the diffusion coefficients of GM1 molecules classified as over (grey) and apart from (magenta) D76N b2M aggregates.

Single particle tracking experiments were performed to confirm the interaction between GM1 and D76N b2M aggregates aged 144 hrs at the plasma membrane of SH‐SY5Y cells. This technique exploits fluorescent semiconductor nanocrystals, QDs, as a very bright and photostable probe to follow the motion of membrane molecules such as lipids and proteins with nanometric resolution in living cells 18. The cells were incubated for 20 min. with aggregates of D76N b2M aged 144 hrs and labelled with anti‐b2M/antimouse Alexa 488 and CTX‐B/QDs 655 (Fig. 5A). At these conditions, D76N b2M aggregates did not diffuse significantly during the recording sessions of 2.5 sec. Then, the GM1 molecules were discriminated according to the localization of their trajectories with respect to the aggregates (Fig. 5B). The linear average MSD plot of GM1 molecules moving across regions away from D76N b2M aggregates reflected a typical Brownian motion (Fig. 5C). By contrast, the GM1 molecules co‐localizing with D76N b2M aggregates displayed a curved average MSD plot that can be ascribed to a confined type of motion (Fig. 5C). Furthermore, the cumulative distributions of diffusion coefficients (D) of single GM1 molecules moving over and separately from D76N b2M aggregates differed substantially (Fig. 5D). The median D value of GM1 molecules overlapping with D76N b2M aggregates was one order of magnitude lower (Table 1). The median D value of freely diffusing GM1 appeared higher than those reported previously 25, 26 1.2 × 10^−1^ μm^2^/sec. with respect to 2.9 × 10^−2^ μm^2^ sec. and 2.7 × 10^−2^ μm^2^/sec. This discrepancy is possibly due to the higher acquisition frame rate used here (100 Hz as compared with 3 Hz). Nevertheless, the absolute value of the change of GM1 D in the presence of D76N b2M aggregates was comparable to that observed in the presence of Aβ1‐42, amylin and Sup35pNM amyloid aggregates 25, 26, 27, 28. Overall, these data show at the single molecule level that the mobility of GM1 in living neuroblastoma cells is altered by b2M aggregates, further supporting the idea that this lipid is a key interaction site even for the aggregates formed by this protein.

**Table 1:** Analysis of GM1 lateral diffusion in the presence or in the absence of D76N b2M aggregates

Condition	*n*	D_median_ (μm^2^/sec.)	Δ_max_ a	*P* b
GM1‐CTX‐B out D76N b2M	33	1.2 × 10^−1^		
GM1‐CTX‐B over D76N b2M	25	2.1 × 10^−2^	6.8 × 10^−1^	≤10^−4^

a Maximum difference in cumulative fraction between GM1‐CTX‐B diffusion over and nearby D76N b2M aggregates.

b Kolmogorov–Smirnov test *P*‐value calculated using Δ_max_ as statistic.

### D76N b2M aggregates interact specifically with synthetic lipid bilayers enriched in GM1

**Figure 6:**
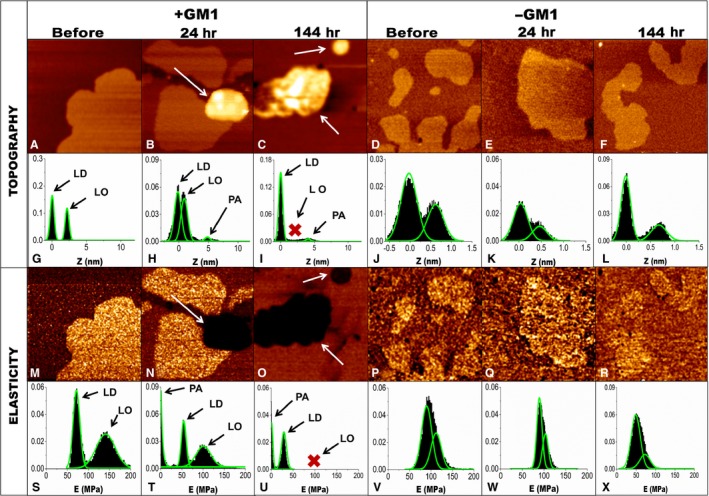
AFM topography and elasticity maps of lipid bilayers obtained from QI mode measurements. Morphology of lipid bilayers enriched in GM1(5%) before the interaction with D76N b2M (**A**) or after the interaction with aggregates of 5.0 μM D76N b2M aged 24 hrs (**B**) or 144 hrs (**C**). The white arrows show accumulated protein aggregates; the red ‘x’ marks the absence of LO phase. The morphology of lipid bilayers lacking GM1 (**D**) was not altered upon interaction with protein aggregates aged 24 hrs (**E**) or 144 hrs (**F**). Height distributions in the presence (**G**–**I**) or in the absence (**J**–**L**) of GM1. Elasticity maps in the presence (**M**–**O**) or in the absence (**P**–**R**) of GM1 and the corresponding Young's modulus distributions (**S**–**U** and **V**–**X**). LO: ordered lipid phase; LD: disordered lipid phase; PA: peak corresponding to the signal arising upon accumulation of aggregates onto LO.

To confirm, and get further insights on the aggregate‐membrane interaction, we also investigated by AFM imaging and elasticity measurements whether aggregate binding affected the physical properties of synthetic lipid bilayers. D76N b2M aggregates were administered to synthetic lipid bilayers with two different lipid mixtures (DOPC:SM:chol:GM1 and DOPC:SM:chol), as described in Materials and Methods. The morphology and elasticity of the bilayers were assessed before and after addition of the aggregates. Both lipid mixtures assembled into a lipid bilayer with two distinct lipid phases. The thicker domains correspond to the ordered lipid phase (LO) that, under our conditions, was enriched in cholesterol, sphingomyelin and GM1, when present. The rest of the sample represents the disordered phase domain (LD), where the DOPC is the main component 30. The difference in eight between LO and LD domains was 2.2 ± 0.6 nm in the presence of GM1 and 0.6 ± 0.3 nm in the absence of GM1 (Fig. 6A and D). Figure 6G and J reports typical height distributions in both cases. The calculation of the elasticity maps revealed that Young's moduli (E) of the two phases were clearly distinct in the sample containing GM1 (Fig. 6M and S), while they were quite similar in the samples without GM1 (Fig. 6P and V). The aggregates grown for 1.0 hr induced a weak destabilization of the bilayers containing GM1 (Fig. S1I and M). Under these conditions, the morphology of the two lipid phases appeared essentially unmodified, the difference in height between the two phases being only slightly changed (2.4 ± 0.4 nm) and the two domains being still clearly distinguishable, yet with the presence of a few small defects (Fig. S1A and E). A global decrease of membrane rigidity was measured in the GM1‐enriched domains compared to the untreated bilayers, where a shift towards reduced values of elasticity was evident (Fig. S1I and M). The aggregates aged 24 hrs induced a more marked perturbation of the membrane structure. Accumulation of material on the LO domains was clearly detected (Fig. 6B, arrow) as a new peak in the height distribution (labelled as PA in Fig. 6). This was not compatible with the formation of a second lipid bilayer on the top of the first one, both in terms of thickness and of elasticity (Fig. 6H, N, T). In particular, the distribution of elasticity confirmed that membrane integrity was still maintained in most of the bilayer area (see peaks LD and LO in Fig. 6T). However, portions of the sample characterized by a significantly lower stiffness (and hence located at lower E values in the distribution in Fig. 6T) were present, and these were associated with the presence of the additional material at the surface of the bilayer, possibly consisting of protein aggregates or complexes formed upon mixing of protein aggregates with sequestered lipids. After the administration of 144‐hr‐aged aggregates, the morphological and structural features associated with the LO domains were completely lost (Fig. 6C and I). Under these conditions, islands of material characterized by a higher roughness and thickness with respect to the intact LO phases were distributed all over the membrane (Fig. 6C). The peak at ≈2.2 nm, characteristic of the LO phase, was completely replaced by the PA peak after the interaction with aggregates aged 144 hrs (Fig. 6I). These domains were also characterized by a low E value (Fig. 6O and U), compatible with that observed with aggregates aged 24 hrs (Fig. 6B). Under these conditions, the elasticity peak associated to the LD phase was still preserved, indicating that the latter was substantially unaffected, possibly due to the weak interaction between protein assemblies and DOPC bilayer. All these findings lend support to the idea that D76N b2M aggregates are recruited onto the LO phase, confirming the importance of GM1 in aggregate recruitment. Substantially superimposable results were observed for aggregates aged 72 hrs (Fig. S1B, F, J and N). To further confirm the specific role played by GM1, the same protein aggregates were administered also to lipid bilayers lacking GM1. In this case, membrane morphology was maintained following administration of aggregates grown for 1 hr (Fig. S1C, G, K and O), 24 hrs (Fig. 6E, K, Q and W), 72 hrs (Fig. S1D, H, L and P) or 144 hrs (Fig. 6F, L R and X). Both the LD and the LO phases were preserved and protein aggregates did not accumulate onto the membrane. In this case, the elasticity analysis was complicated by the overlap between the E distributions associated with the two phases. Nevertheless, a global decrease of stiffness was detected as a consequence of GM1 depletion, but the shape of the distribution was essentially unaltered following addition of the aggregates, indicating the absence of selective perturbations in either phase.

## Discussion

D76N b2M is the first described genetic variant of b2M associated with a familial form of systemic amyloidosis. The protein displays a stronger amyloidogenic propensity with respect to the wild‐type form and aggregates easily *in vitro* in PBS. The determination of the three‐dimensional structure and the mutation‐related behaviour of this variant have provided valuable information about the molecular basis of D76N b2M aggregation 4, 10. In spite of this information and the interest raised by the discovery of this b2M variant in the population, little is known about its aggregation path, its ability to interact with synthetic and biological membranes and the molecular mechanisms of aggregate cytotoxicity. In the present study, we aimed to provide knowledge on these issues by exploiting a battery of complementary biophysical techniques and cell biology methods.

It has been long recognized that the unstable, heterogeneous pre‐fibrillar assemblies transiently populated during the early phases of *in vitro* aggregation of many different proteins are the main culprit of amyloid‐related toxicity 31. Conversely, although the fibrils are considered stable, they can still act as reservoirs of toxic species 32, 33. Furthermore, when such species are added to cultured cells, their specific interaction with the cell membrane could induce fibril fragmentation leading to new or enhanced, deleterious, biological responses; similar findings were previously reported for wild‐type b2M 33, 34. In addition, aberrant surface properties of amyloid species may influence their toxicity; these include the presence of hydrophobic patches that may act as sticky sites promoting aberrant interaction of amyloid assemblies with the cells or, alternatively, aggregate clumping. Our DLS and TEM analysis showed that under our conditions, D76N b2M fibrillar aggregates aged 144 hrs were the most toxic species, while aggregates obtained at earlier incubation times were composed of non‐fibrillar, spheroidal species with a high tendency to associate and to form clumps. We found that the fluorescence intensity of ANS in the presence of these early species was higher than that recorded in the presence of the 144‐hr‐aged cytotoxic assemblies. Given that all the samples aged up to 24 hrs exhibited the same content of β‐structure, as assessed by ThT, CR and CD spectra, indicating an already established amyloid‐like conformation (Fig. 1A), the hydrophobic side chains of the aged samples were expected to be less accessible to ANS than those of samples aggregated for shorter times, presumably due to a different packing of the protofilaments. When moved to a cell growth medium, these fibrillar samples interacted with the cell membrane, preferentially with negatively charged species such as the sialic acid of GM1, suggesting that such interaction does not involve hydrophobic patches but, rather, electrostatic interactions.

It is likely that the interaction between aggregates obtained after 144 hrs of incubation and membrane surfaces results in membrane damage and cytotoxicity through a mechanism involving the release of cytotoxic species, as hypothesized elsewhere 35. This idea was corroborated by a poorly scattered signal around larger D76N b2M aggregates in the FRET analysis.

The observations arising from our study raise the possibility that the toxicity of protein aggregates is not necessarily related to the oligomeric nature but rather to structural and surface properties that may or may not be shared by early and late aggregates. Our findings showed that differently from many other aggregating peptides and proteins, the mature aggregates of D76N b2M displayed the most severe cytotoxic effects, in agreement with the damage induced by wild‐type b2M fibrils 36, whereas the aggregation intermediates, including early aggregates, commonly considered most cytotoxic, were indeed less cytotoxic. This finding, albeit unusual, is not entirely surprising since in several cases fibrils toxicity eventually culminating with necrotic cell death, as in our case, has been reported 37, 38, 39. Our toxicity results were coherent with the other investigated features of the D76N b2M pre‐fibrillar aggregates and fibrils, including their ability to interact with synthetic and biological membranes. When incubated with cells, these samples interacted with the cell membrane preferentially in regions enriched in GM1, possibly through its negatively charged sialic acid residue, as shown for many other amyloid systems 25, 26. This suggested that such interaction was mediated predominantly by electrostatic interactions. The key role played by GM1 in aggregate recruitment was also confirmed by our FRET experiments, showing a close proximity between D76N b2M aggregates and GM1 on the cell membrane. In addition, QD experiments evidenced a remarkable alteration of the type of motion of GM1 on the cell membrane in the absence (Brownian) or in the presence (confined) of the aggregates, further confirming the strict relationship between aggregates and GM1 and adding to a huge body of studies that support the importance of GM1 not only as a key aggregate nucleation site 40, 41 but also as a major aggregate interaction site on the cell membrane 42. Considering that biochemical modifications such as alterations of intracellular free Ca^2+^ and ROS levels are amongst the earliest modifications associated with the membrane perturbations that underlie cell suffering in aggregate‐exposed cells, our data further support the establishment of GM1‐aggregate interaction as a key switch of amyloid cytotoxicity. Our measurements with AFM in QI mode on model membrane reconstructed on a mica surface further support previous results and, in addition, confirm GM1 interaction as an event required for cytotoxicity. Furthermore, these data point to possible toxicity mechanisms based on a perturbation of the elasticity (E) of specific (GM1 enriched) ordered lipid phases (LO) following aggregate interaction with the cell membrane, as observed for other amyloid‐related damaging systems 43, 44. Such results also indicate that there is a clear progression in the capability of D76N b2M aggregates to damage synthetic membranes after 1, 24, 72 and 144 hrs of aggregation, the effect being not observable after 1 hr, significant just after 24 and maximal at 72 and 144 hrs. Although the evaluation of E for extremely thin layers of soft materials lying on rigid substrates is still considered a challenging task, we obtained extremely reproducible results, which also agree with those obtained, by means of a similar approach, by Picas and co‐workers 45.

In conclusion, our data provide a first glimpse on the aggregation path, membrane interaction and cytotoxicity of the only known b2M variant associated with a familial form of systemic b2M amyloidosis. Further investigations will define more precisely the molecular and environmental players affecting D76N b2M aggregation in tissues, the molecular details and the biochemical and cell biology modifications that underlie the physiological effects of those species. When added to those pertaining wild‐type b2M, these data will aid depicting more precisely the scenario of b2M deposition, providing clues for the search of molecules and treatments aimed at tackling the pathologies associated with this phenomenon.

## Conflicts of interest

The authors confirm that there are no conflicts of interest.

## Supporting information


**Figure S1** AFM topography and elasticity maps of lipid bilayers obtained from QI mode measurements. Morphology of lipid bilayers enriched in GM1 (5%) in presence of aggregates of 5.0 μM D76N b2M aged 1 hr (**A**) or 72 hrs (**B**). The white arrows show accumulated protein aggregates; the red ‘x’ marks the absence of LO phase. The morphology of lipid bilayers lacking GM1 was not altered upon interaction with protein aggregates aged 1.0 hr (**C**) or 72 hrs (**D**). Height distributions in the presence (**I** and **J**) or in the absence (**K** and **L**) of GM1. Elasticity maps in the presence (**M** and **N**) or in the absence (**O** and **P**) of GM1 and the corresponding Young's modulus. LO: ordered lipid phase; LD: disordered lipid phase; PA: peak corresponding to the signal arising upon accumulation of aggregates onto LO.

**Figure S2** Cytotoxicity of D76N b2M aggregates on HL‐1 cells. (**A** and **B**) HL‐1 cells exposed for 24 hrs to 5.0 μM D76N b2M aggregated for 24 hrs (**A**) and 144 hrs (**B**). The cells were stained with Alexa 488‐conjugated CTX‐B (green fluorescence); protein aggregates were stained with anti‐b2M antibodies followed by treatment with Alexa 568‐conjugated anti‐rabbit secondary antibodies (red fluorescence). FRET efficiency is shown in panels 1, 2 for aggregates aged 24 hrs or 144 hrs, respectively. (**C**) MTT assay on HL‐1 cells exposed for 24 hrs to 5.0 μM D76N b2M samples aggregated for different times. (**D**) ROS production in HL‐1 cells exposed for 24 hrs to D76N samples (5.0 μM) aggregated for varying lengths of time. Error bars in all bar plots indicate the standard deviation of three independent experiments carried out in triplicate. *T*‐test analysis: *P < 0.005; **P < 0.001;***P < 0.0001 *versus* untreated cells.
