# Supplementary figures and images for: Molecular insights into cell toxicity of a novel familial amyloidogenic variant of β2‐microglobulin
Source: J Cell Mol Med. 2016 Mar 18;20(8):1443–56. doi: 10.1111/jcmm.12833 (PMC4956941; doi:10.1111/jcmm.12833)

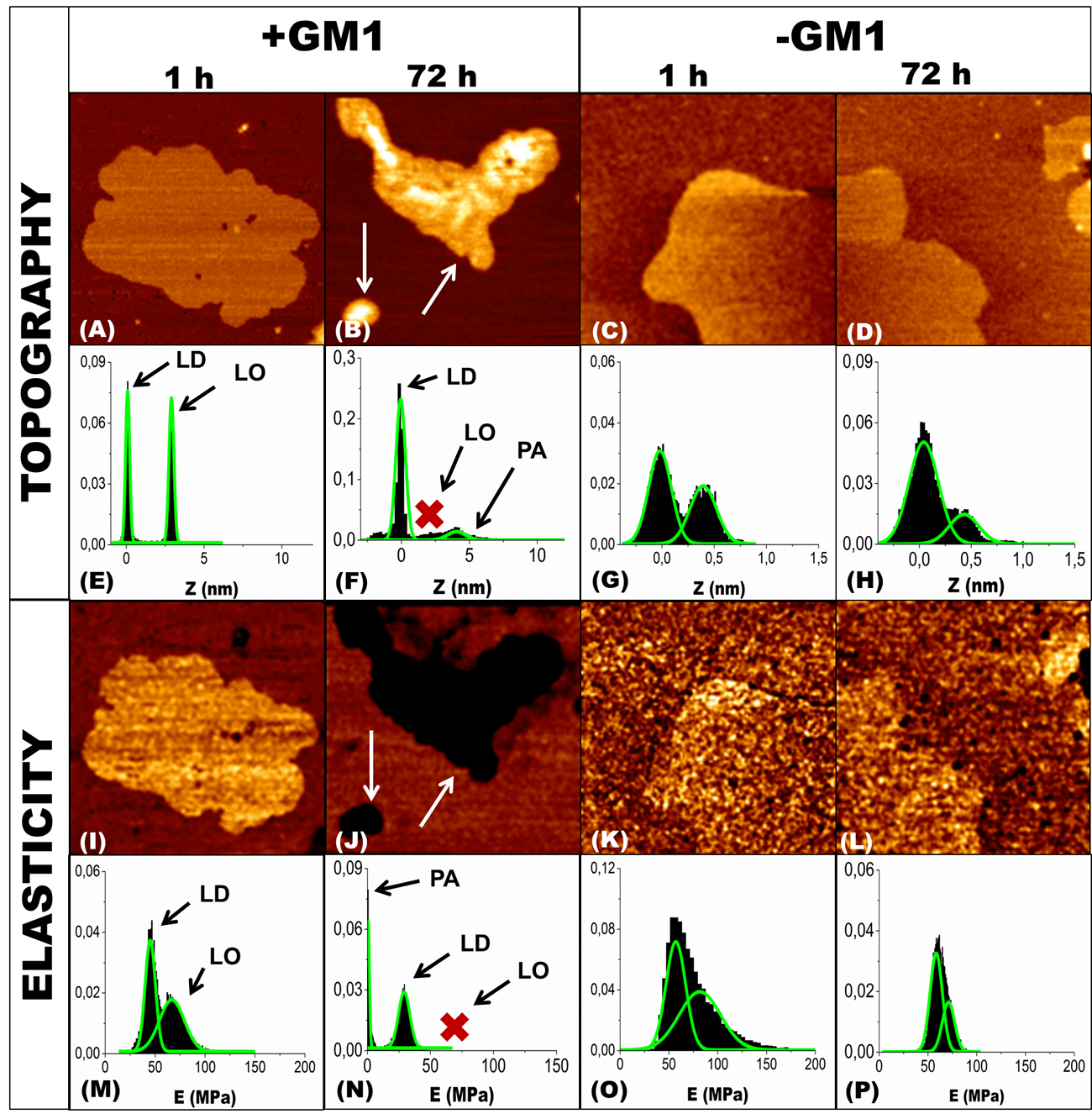

Supplement: Supplementary file 1 — Figure S1 AFM topography and elasticity maps of lipid bilayers obtained from QI mode measurements. Morphology of lipid bilayers enriched in GM1 (5%) in presence of aggregates of 5.0 μM D76N b2M aged 1 hr (A) or 72 hrs (B). The white arrows show accumulated protein aggregates; the red ‘x’ marks the absence of LO phase. The morphology of lipid bilayers lacking GM1 was not altered upon interaction with protein aggregates aged 1.0 hr (C) or 72 hrs (D). Height distributions in the presence (I and J) or in the absence (K and L) of GM1. Elasticity maps in the presence (M and N) or in the absence (O and P) of GM1 and the corresponding Young's modulus. LO: ordered lipid phase; LD: disordered lipid phase; PA: peak corresponding to the signal arising upon accumulation of aggregates onto LO. [file JCMM-20-1443-s001.tif]

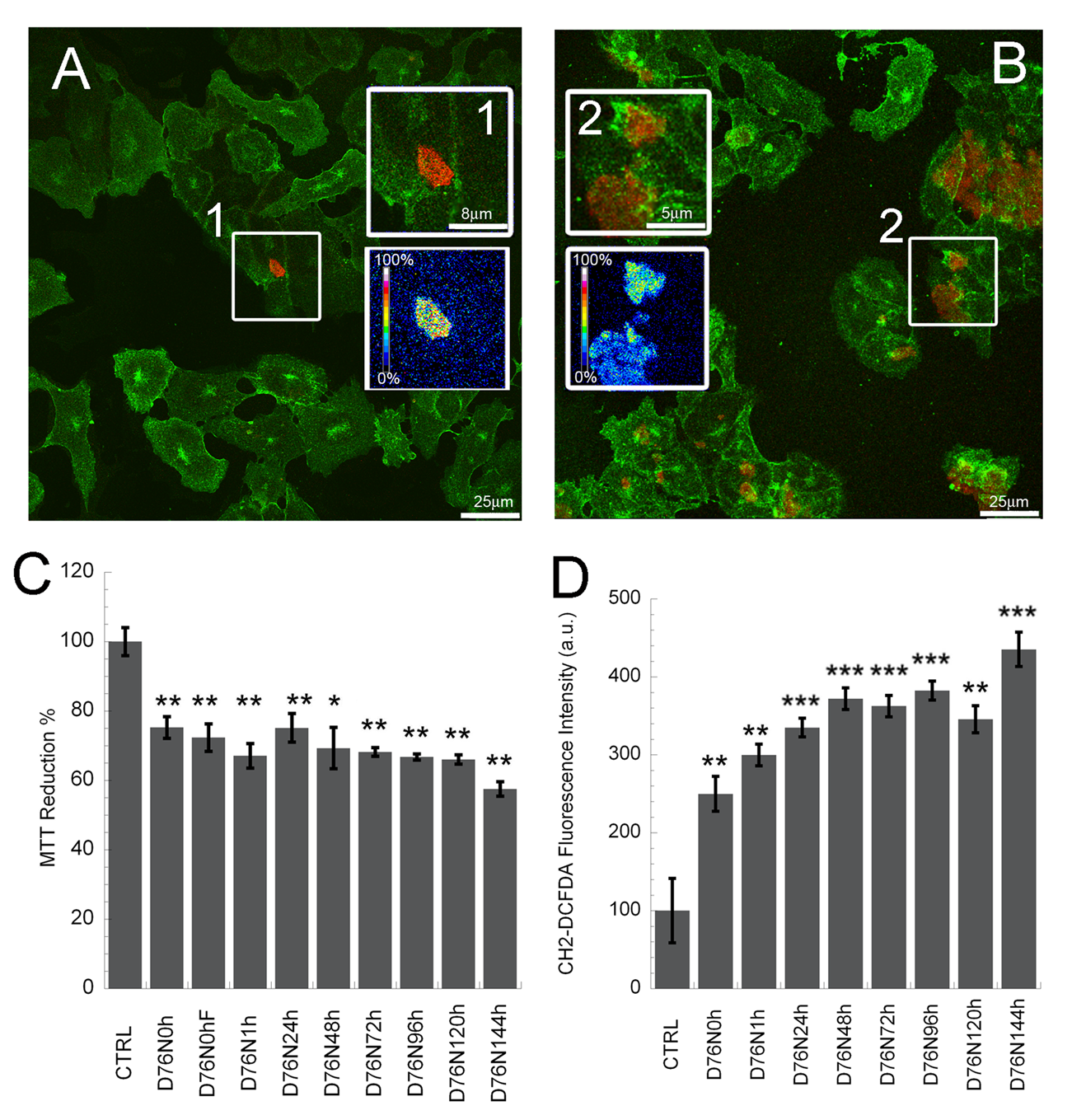

Supplement: Supplementary file 2 — Figure S2 Cytotoxicity of D76N b2M aggregates on HL‐1 cells. (A and B) HL‐1 cells exposed for 24 hrs to 5.0 μM D76N b2M aggregated for 24 hrs (A) and 144 hrs (B). The cells were stained with Alexa 488‐conjugated CTX‐B (green fluorescence); protein aggregates were stained with anti‐b2M antibodies followed by treatment with Alexa 568‐conjugated anti‐rabbit secondary antibodies (red fluorescence). FRET efficiency is shown in panels 1, 2 for aggregates aged 24 hrs or 144 hrs, respectively. (C) MTT assay on HL‐1 cells exposed for 24 hrs to 5.0 μM D76N b2M samples aggregated for different times. (D) ROS production in HL‐1 cells exposed for 24 hrs to D76N samples (5.0 μM) aggregated for varying lengths of time. Error bars in all bar plots indicate the standard deviation of three independent experiments carried out in triplicate. T‐test analysis: *P < 0.005; **P < 0.001;***P < 0.0001 versus untreated cells. [file JCMM-20-1443-s002.tif]
